# Anticancer Activity and Mode of Action of Cu(II), Zn(II), and Mn(II) Complexes with 5-Chloro-2-*N*-(2-quinolylmethylene)aminophenol

**DOI:** 10.3390/molecules28124876

**Published:** 2023-06-20

**Authors:** Shuangshuang Gai, Liqin He, Mingxian He, Xuwei Zhong, Caiyun Jiang, Yiming Qin, Ming Jiang

**Affiliations:** Key Laboratory for Zhuang and Yao Pharmaceutical Quality Biology, School of Food and Biochemical Engineering, Guangxi Science & Technology Normal University, Laibin 546199, China; gaishuangshuang@gxstnu.edu.cn (S.G.); 13397802652@163.com (L.H.); hemingxian@gxstnu.edu.cn (M.H.); zhongxuwei@gxstnu.edu.cn (X.Z.); jiangcaiyun@gxstnu.edu.cn (C.J.)

**Keywords:** anticancer, DNA, metal complexes, lung cancer

## Abstract

Developing a new generation of anticancer metal-based drugs that can both kill tumor cells and inhibit cell migration is a promising strategy. Herein, we synthesized three Cu(II), Zn(II), and Mn(II) complexes derived from 5-chloro-2-*N*-(2-quinolylmethylene)aminophenol (C1–C3). Among these complexes, the Cu(II) complex (C1) showed significantly greater cytotoxicity toward lung cancer cell lines than cisplatin. C1 inhibited A549 cell metastasis and suppressed the growth of the A549 tumor in vivo. In addition, we confirmed the anticancer mechanism of C1 by triggering multiple mechanisms, including inducing mitochondrial apoptosis, acting on DNA, blocking cell cycle arrest, inducing cell senescence, and inducing DNA damage.

## 1. Introduction

Cisplatin is widely used to treat various solid tumors; it can induce cancer cell apoptosis by acting on DNA and results in cancer cell death [1,2]. However, the clinical application of cisplatin is limited because of the following factors: (1) easy-to-develop drug resistance [3,4]; (2) severe side effects on organs [1,5,6]; and (3) stimulating metastasis of cancer [7,8,9]. Accordingly, current anticancer metal-based drug research focuses on developing compounds with other metal centers, which may be a promising strategy.

Currently, to develop the next generation of metal drugs and realize effective anticancer treatment, Cu, Ru, Zn, Au, Mn, and other metal complexes have been widely synthesized for tumor therapy, which may exhibit different anticancer functions and specific patterns [10,11,12,13,14]. Among them, Cu, Zn, and Mn are essential trace elements in the human body, which are vital in maintaining human health and physiological activities [15,16,17]. Furthermore, Cu, Zn, and Mn complexes strongly inhibit cancer cell growth and have high selectivity [14,18,19].

The Schiff base is a condensation product of primary amine and aldehyde (or ketone) compounds, in which the resulting azomethine nitrogen atoms have very important biological activities, including antitumor properties [20,21,22,23]. Aminophenol Schiff base ligands have recently gained extensive attention because they have good antitumor activity [24,25,26,27], and they possess strong coordination ability and diverse coordination modes owing to their oxygen and nitrogen atoms coordination sites [28,29]. Interestingly, several metal complexes have also been synthesized using aminophenol Schiff base ligands and have shown remarkable antitumor activity [24,28,30]. However, there still remain mysteries about their antitumor mechanism. Therefore, we proposed to design a novel non-Pt compound based on 5-chloro-2-*N*-(2-quinolylmethylene) aminophenol Schiff base ligand, which may be a promising approach to enhance selectivity.

Considering the above factors, we designed and synthesized a series of non-Pt (Cu^II^, Zn^II^, and Mn^II^) complexes (C1, C2, and C3, respectively) derived from 5-chloro-2-*N*-(2-quinolylmethylene) aminophenol Schiff base ligand and assessed their anticancer activity. Among them, the Cu complex (C1) exhibited the highest anticancer activity. Subsequently, we investigated the antimetastasis ability of C1 and confirmed the mechanism of C1 multi-actions on lung cancer cells. Finally, we further evaluated the antitumor activity of C1 in mice bearing A549 tumor. Our results provide new insights to develop novel non-Pt complexes with potent antitumor and antimetastasis activities.

## 2. Results and Discussion

### 2.1. Synthesis and Characterization of Cu(II), Zn(II), and Mn(II) Complexes

**Scheme 1:**
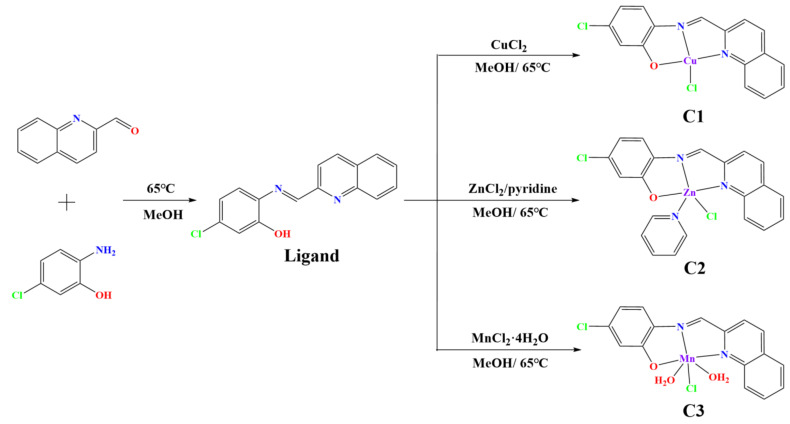
Synthetic route for C1–C3.

**Figure 1:**
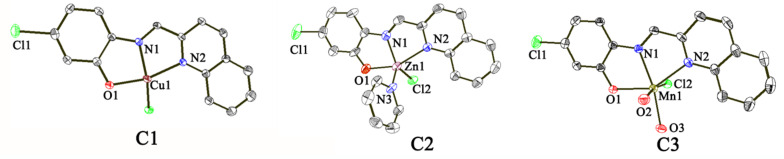
Crystal structures of Cu(II), Zn(II), and Mn(II) complexes (C1–C3).

The Schiff base ligand (L) was formed by reacting 2-quinolinecarboxaldehyde with 5-chloro-2-aminophenol in methanol. Subsequently, the corresponding Cu(II), Zn(II), and Mn(II) complexes were synthesized in situ (Scheme 1). Single crystals of Cu(II), Zn(II), and Mn(II) complexes were determined using X-ray crystallography and solved using Olex 2.0 software. The molecular structures of Cu(II), Zn(II), and Mn(II) complexes (C1–C3) are shown in Figure 1. The crystallographic data of these complexes are reported in Tables S1 and S2.

C1 crystallizes in the monoclinic system with the space group P2_1_/c. The coordination geometry of Cu^2+^ is square planar with one quinoline-N [Cu1–N2 = 2.076(3) Å], one imine-N [Cu1–N1 = 1.944(3) Å], one phenolate-O [Cu1–O1 = 1.959(3) Å] from ligand, and one Cl atom [Cu1–Cl1 = 2.2479(11) Å].

C2 crystallizes in the triclinic system with the P-1 space groups. The center Zn^2+^ adopts a square pyramidal distortion. Two N and one O atoms donated by the Schiff base ligand (N1, N2, and O1) and one Cl atom (Cl2) form a plane. The other N (N3) from pyridine is positioned at the vertex.

C3 crystallizes in the triclinic system with the space group P-1; the distorted octahedral coordination sphere around the Mn^2+^ is assembled from the N,N,O-tridentate ligand, two H_2_O ligands, and one Cl^−^ ion.

The structures of L and C1–C3 were further characterized (Figures S1–S12). In the IR spectra of L, the peaks at 2315 and 1796 were attributed to the ν_C=N_ stretching bands; the peak at 3736 cm^−1^ corresponded to the stretching vibrations of phenolic ν_O–H_. When L formed complexes (C1–C3) with metal ions, the peak of ν_O–H_ disappeared and the peaks of C=N were shifted. The complexes were confirmed by comparing the IR spectra of C1–C3 and L. In addition, the ^1^H-NMR and ^13^C-NMR spectra of L (Figure S1) and C2 were measured and were found to be consistent with the expected structures.

### 2.2. The Solubility and Stability of Cu(II), Zn(II), and Mn(II) Complexes in Saline

In order to carry out the following biological activity assays, we tested the solubility of the metal complexes in saline (0.9% sodium chloride solution). The solubility of C1, C2, and C3 was ≥1.9, 1.3, and 0.8 mg/mL, respectively. These results indicated C1–C3 had good solubility in saline. Then, we further evaluated the stability of C1–C3 in saline by UV-vis spectroscopy. As shown in Figure S13, there were no significant changes of absorbance spectra for 48 h, indicating that C1–C3 were stable in saline for 48 h. Therefore, C1–C3 were dissolved in saline for subsequent cell assays.

### 2.3. The Cytotoxicity of Cu(II), Zn(II), and Mn(II) Complexes In Vitro

The cytotoxicity of C1–C3 was determined using an MTT (3-(4,5-dimethylthiazol-2-yl)-2,5-diphenyltetrazolium bromide) assay on various cancer cells. As shown in Table 1, the IC_50_ values of the Schiff base ligand were significantly higher than those of the metal complexes. The Cu complex (C1) exerted the highest cytotoxic potency compared with the Zn(II) and Mn(II) complexes in A549 cells, with IC_50_ values of 3.93 μM, while IC_50_ values of C2 and C3 were 18.26 and 33.61 μM, respectively. In addition, C1 had the most potent activity against cisplatin-resistant lung cancer (A549cisR) cells. Notably, compared to cisplatin, Cu complexes not only had superior anticancer activity, but also exhibited better selectivity. Therefore, C1 was selected to carry out the following studies.

### 2.4. Inhibition of C1 on Migration and Invasion Activities

**Figure 2:**
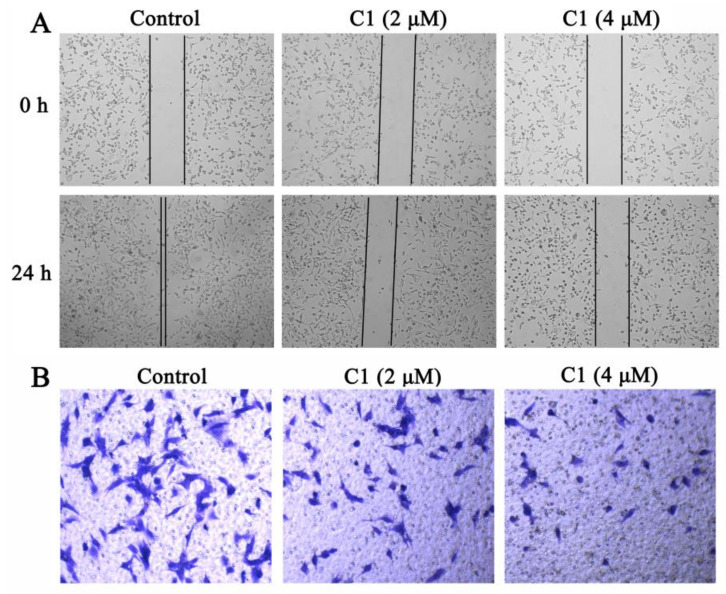
(**A**) The anti-migratory effect of C1 on A549 cells by wound-healing assay. (**B**) A549 cells’ invasion ability with C1 treatment for 24 h in transwell assays.

Cell migration is crucial to cancer development and metastasis [31,32]; metastasis is a major cause of many cancer-related deaths [33,34]. The migration is very usually assessed by the application of the wound-healing assay, which involves the creation of an artificial gap in a cancer cell monolayer, followed by observation of the area covered with migrating cells [35]. Therefore, the inhibiting effect of C1 on cancer cell migration was evaluated using a wound-healing assay. Figure 2A shows that the significant migration of A549 cells to the blank area in the control group compared with that in the C1 group, indicating that C1 strongly inhibited the migration ability of A549 cells. In addition, the effect of C1 on A549 cell invasion was investigated using a transwell assay. The number of cells that invaded across the inserts was significantly lower in the C1-treated group than in the untreated group (Figure 2B), indicating that C1 could inhibit the invasion abilities of A549 cells.

### 2.5. Mechanism of Action of C1

#### 2.5.1. Inducing Mitochondrial-Mediated Apoptosis

**Figure 3:**
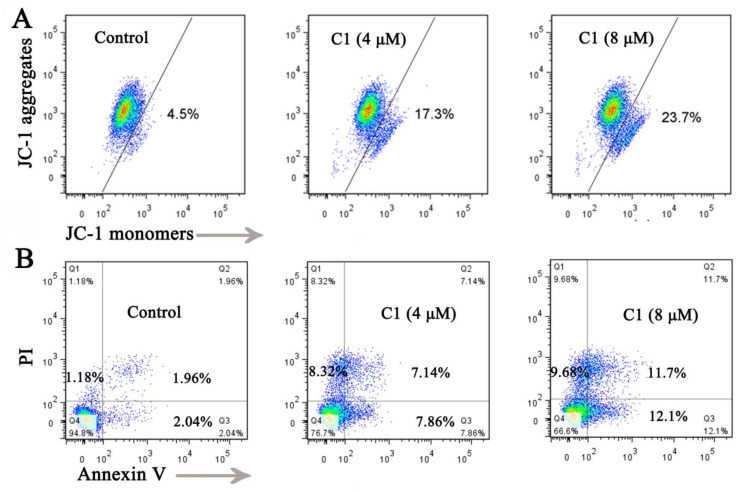
(**A**) JC-1 staining of C1-treated A549 cells detected using flow cytometry. (**B**) The effect of C1 on A549 cells apoptosis after treatment with C1 for 24 h.

Mitochondria play a key role in death signals through intrinsic and extrinsic apoptosis [36,37]. Therefore, after exposure to C1, A549 cells were stained with a JC-1 probe to evaluate the mitochondrial membrane potential (ΔΨm) using flow cytometry. The results from Figure 3A showed that the ΔΨm of A549 cells was reduced by 17.3% and 23.7% when treated with C1 at concentrations of 4 and 8 μM, respectively, strongly indicating that C1 altered ΔΨm and induced mitochondrial dysfunction.

Then, the effect of C1 on A549 cells apoptosis was stained with Annexin V/PI and analyzed using flow cytometry. Figure 3B indicates that when A549 cells were treated with C1, the population of apoptotic cells considerably increased (15.0% and 23.8% for 4 and 8 μM, respectively) compared to the control group (4.0%), suggesting that C1 induced apoptosis in A549 cells.

#### 2.5.2. DNA Binding

**Figure 4:**
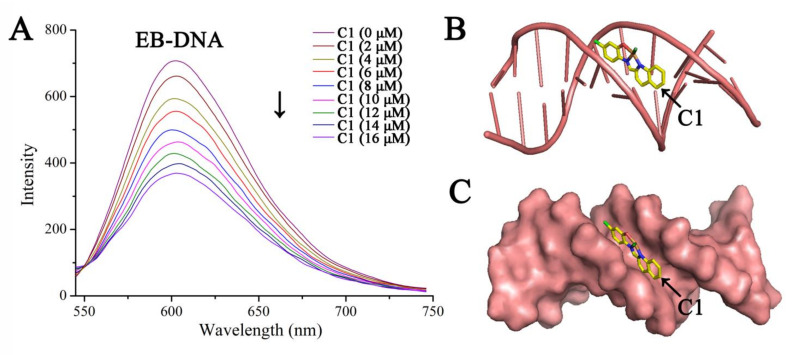
(**A**) The emission spectra of EB-DNA with successive addition of C1. (**B**,**C**) Docked pose of Cu complex and DNA.

Competitive studies with ethidium bromide (EB) have been widely used in fluorescence-quenching assays to evaluate the interaction between DNA and small molecules [38]. Therefore, to further explore the binding property of C1 and DNA, fluorescence quenching was carried out to explore the interaction mode. As shown in Figure 4A, the EB-DNA solution emits strong fluorescence at 550–750 nm, and the maximum fluorescence appears at 601 nm. As the C1 content increased, a decrease in the emission intensity was observed, indicating that C1 exhibited better competitive ability than EB and displaced EB from the hydrophobic pockets of DNA. These results verified that C1 binds to DNA in an intercalative binding mode.

Next, docking studies were conducted to investigate the interaction between C1 and DNA. According to our docking calculations, the docked binding energy value was −7.63 kcal/mol. As shown in Figure 4B,C, the Cu complex fit well into the curved profile in a minor groove of DNA (PDB ID: 1D64), and formed hydrogen bonds with surrounding bases. As a result, C1 can be stably intercalated in the double helix DNA strand in a similar mode to similar aminophenol Schiff base derivatives [39].

#### 2.5.3. Arresting Cell Cycle

**Figure 5:**
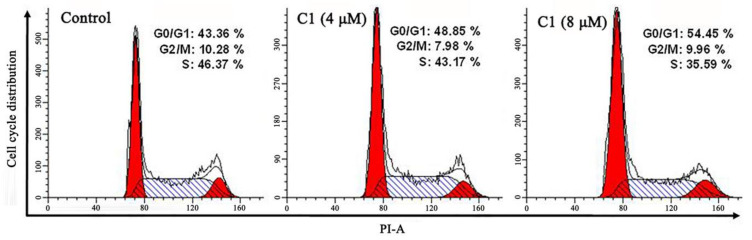
The effect of C1 on the cell cycle in A549 cells after treatment for 48 h, analyzed using flow cytometry.

The influence of metal compounds on cell cycle arrest contributes to their anticancer activity [40]. To determine the effect of C1 on the cell cycle, the distribution of the cell cycle of C1-treated A549 cells was detected using flow cytometry. As shown in Figure 5, after treatment with 4 and 8 μM C1 for 48 h, the percentage of cells in the G1/G0 phase increased to 48.85% and 54.45%, respectively. This result indicates that C1 can block the A549 cell cycle in the G1/G0 phase.

#### 2.5.4. Inducing A549 Cell Senescence

**Figure 6:**
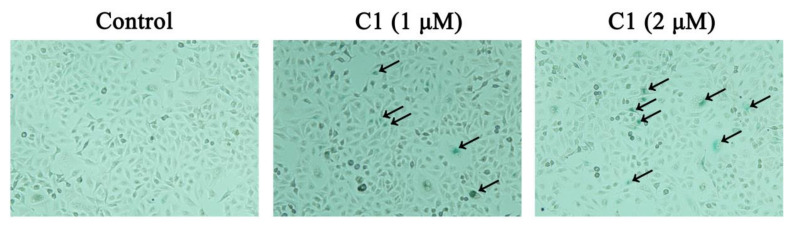
Cell senescence after treatment with C1, studied using a β-galactosidase staining assay (senescent cells marked by black arrows).

Senescence is a permanent cellular stage involving cell cycle arrest, which is a potential strategy against cancer [41]. We measured the impact of C1 on cell senescence in A549 cells using a β-galactosidase staining assay. The senescent cells were dyed blue by β-galactosidase. As shown in Figure 6, the number of blue-stained cells was significantly higher in the C1 group compared to that observed in the control group, and the C1-treated cells were accompanied by noticeable morphological changes. These results suggested that C1 can induce A549 cell senescence.

#### 2.5.5. Inducing DNA Damage

**Figure 7:**
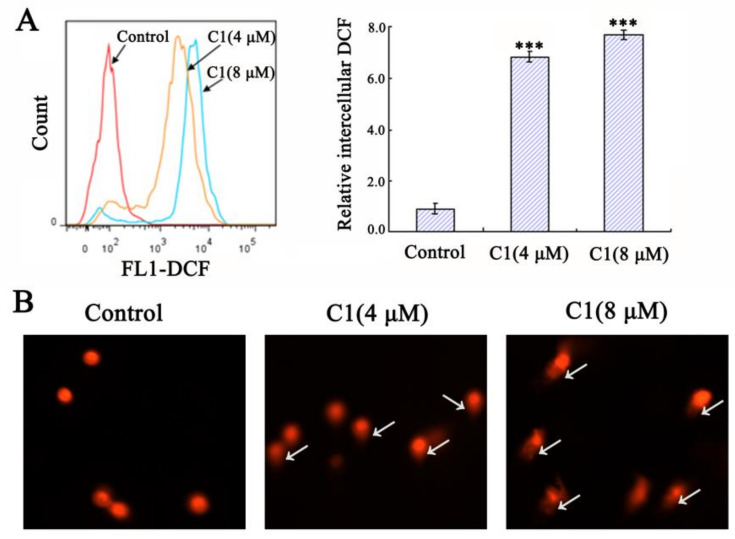
(**A**) Flow cytometry analysis of ROS level in C1-treated A549 cells. *** *p* < 0.001. (**B**) DNA fragmentation triggered by C1 in A549 cells, measured using a comet assay (the comet tails marked with white arrows).

A change in the mitochondrial membrane potential results in mitochondrial dysfunction, which can induce reactive oxygen species (ROS) production [42]. Based on the above information, we detected the ROS level in A549 cells using flow cytometry. As shown in Figure 7A, the intracellular ROS was significantly increased after treating the cells with C1 compared to the control.

Excessive intracellular ROS can cause DNA damage and cell death [43,44]. Next, a comet assay was performed to determine whether C1 caused DNA damage. Undamaged DNA cannot migrate, and fluorescence is only confined to the nucleus. In DNA-damaged cells, negatively charged DNA fragments are then released from the nucleus and migrate towards the anode. There was no comet-like cell observed in the control group. In comparison, C1-treated cells with longer tails (white arrows) were observed, and a higher percentage, indicating the existence of severe DNA fragmentation (Figure 7B). These results strongly suggest that C1 induces DNA damage.

### 2.6. In Vivo Antitumor Efficacy of C1

**Figure 8:**
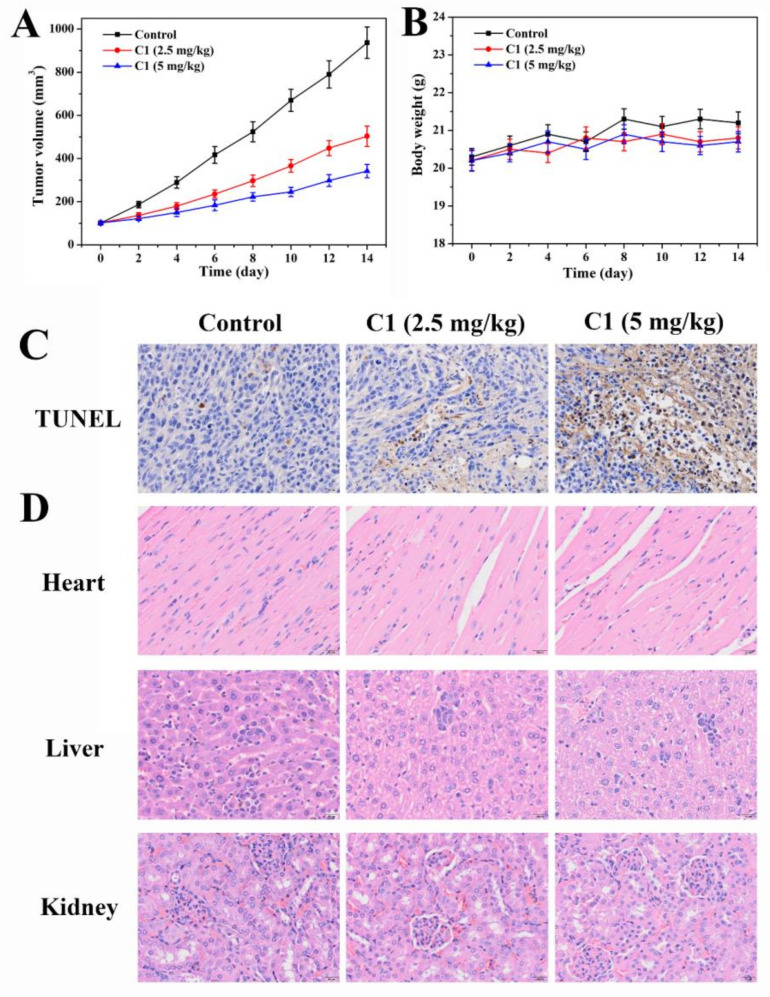
(**A**) Tumor growth inhibition curves of mice treated with C1. (**B**) The body weight of the mice throughout the follow-up period in the treatment of C1. (**C**) TUNEL staining of tumor tissues. (**D**) H&E staining of the major organs (heart, liver, and kidney).

Based on these promising biological properties, the in vivo therapeutic efficacy of C1 was further investigated in mice bearing A549 tumors. As displayed in Figure 8A, the tumors grew rapidly in untreated mice. The mice treated with a dose of C1 at 2.5 mg/kg showed slight inhibitory effect on tumor growth, with an inhibition rate of 46.3%. However, the mice injected with a high dose of C1 at 5 mg/kg showed a high therapeutic efficacy (63.5%). Generally, body weight change is considered a key indicator of systemic toxicity. Indeed, no apparent weight loss (Figure 8B) was observed in mice treated with C1 compared to the control group.

In addition, TUNEL (terminal deoxynucleotidyl transferase dUTP nick end labeling) staining of tumor tissue sections was performed to examine the effect of C1 on apoptosis in vivo. As shown in Figure 8C, the group treated with C1 exhibited notable cell apoptosis, whereas the control group had few apoptotic cells. Histological analyses were carried out using H&E (hematoxylin and eosin) staining to evaluate the toxicity of C1 for the major organ. As shown in Figure 8D, no pathological alterations or other abnormalities were detected in the histological analyses of the heart, liver, and kidney after treatment with C1, indicating that C1 exhibited biocompatibility and biosafety in vivo.

## 3. Materials and Methods

### 3.1. Reagents and Materials

All chemicals were purchased from InnoChem Company (Beijing, China) or Sigma-Aldrich Company (St. Louis, MO, USA). All cell lines were supplied by the Shanghai Institute for Biochemistry and Cell Biology (Shanghai, China). The cells were cultured at 37 °C in 5% CO_2_. Elemental analyses (C, N, and H) were carried out on a Perkin-Elmer 2400 analyzer. Mass spectra of each fragment were measured on a Bruker micrOTOF-Q II. Infrared (IR) spectra were recorded using KBr pellets (4000–400 cm^−1^) on a Nexus 870 FT-IR spectrophotometer.

### 3.2. Synthesis of Cu, Zn, and Mn Complexes

The ligands were prepared according to the procedure we reported previously [45]. A mixture of 2-quinolinecarboxaldehyde (3 mmol) and 5-chloro-2-aminophenol (3 mmol) was dissolved in MeOH (20 mL) and heated at 65 °C. After 4 h, the solvent was eliminated under vacuum, then washed with methanol and dried to obtain a yellow powdered ligand.

5-chloro-2-*N*-(2-quinolylmethylene) aminophenol (L). Yield: 89.7%. Elemental analysis for C_16_H_11_ClN_2_O: C, 67.97; H, 3.92; N, 9.91; O, 5.66. Found: C, 66.79; H, 3.95; N, 9.98; O, 5.74. ^1^H NMR (400 MHz, DMSO-*d6*) δ 9.89 (s, 1H), 8.91 (s, 1H), 8.58–8.47 (m, 2H), 8.13 (d, *J* = 8.4 Hz, 1H), 8.07 (d, *J* = 8.2 Hz, 1H), 7.89–7.80 (m, 1H), 7.74–7.67 (m, 1H), 7.43 (d, *J* = 8.5 Hz, 1H), 7.03 (d, *J* = 2.3 Hz, 1H), 6.94 (d, *J* = 10.8 Hz, 1H). ^13^C NMR (101 MHz, DMSO-*d6*) δ 160.50, 155.24, 153.02, 147.81, 137.19, 136.30, 132.56, 130.62, 129.67, 128.95, 128.54, 128.35, 121.85, 119.97, 119.23, 116.70. IR spectrum of L1. IR (KBr) peaks: 3736 (ν_O–H_), 2315, 1796 (ν_C=N_), 1582, 1500, 1428, 1313, 1223 (ν_C–N_), 832, 754, and 679 cm^−1^. ESI-MS: 281.18 [M − H].

Then, 0.1 mmol ligand and 0.1 mmol CuCl_2_/ZnCl_2_ (containing 1 mL pyridine)/MnCl_2_·4H_2_O were dissolved in MeOH (20 mL) and refluxed at 65 °C for 4 h. The resulting solution was volatilized naturally at room temperature, and the crystals of Cu, Zn, and Mn complexes were obtained after a week.

5-chloro-2-*N*-(2-quinolylmethylene)aminophenoxide-Cu(II)-chloride(C1). Yield: 73.5%. Solubility in saline: ≥1.9 mg/mL. Melting points: 242 °C. Elemental analysis for C_16_H_10_Cl_2_CuN_2_O: C, 50.48; H, 2.65; N, 7.36; O, 4.20. Found: C, 50.66; H, 2.59; N, 7.31; O, 4.23. IR spectrum of C1. IR (KBr) peaks: 2310, 1795 (ν_C=N_), 1523, 1243, 1179, 1053 (ν_C–N_), 843, 749 and 689 cm^−1^. ESI-MS: 380.00 [M + H]. CCDC No. 1436726.

5-chloro-2-*N*-(2-quinolylmethylene)aminophenoxide-Zn(II)-chloride/pyridine (C2). Yield: 69.8%. Solubility in saline: ≥1.3 mg/mL. Melting points: 217 °C. Elemental analysis for C_21_H_15_Cl_2_N_3_OZn: C, 54.64; H, 3.28; N, 9.10; O, 3.47. Found: C, 54.79; H, 3.25; N, 9.13; O, 3.40. ^1^H NMR (400 MHz, DMSO-*d6*) δ 8.93 (s, 1H), 8.61 (d, *J* = 8.5 Hz, 1H), 8.57–8.52 (m, 2H), 8.26–8.24 (m, 2H), 8.16 (d, *J* = 8.5 Hz, 1H), 8.09 (d, *J* = 7.2 Hz, 1H), 7.89–7.84 (m, 1H), 7.74–7.70 (m, 1H), 7.45 (d, *J* = 8.5 Hz, 1H), 7.29 (d, *J* = 8.5 Hz, 2H), 7.05 (d, *J* = 2.3 Hz, 1H), 6.97–6.94 (m, 1H). ^13^C NMR (101 MHz, DMSO-*d6*) δ 161.68, 157.86, 156.44, 153.47, 148.30, 144.30, 137.73, 136.70, 132.98, 131.33, 131.15, 129.94, 129.26, 128.74, 128.43, 122.37, 120.47, 119.38, 117.19. IR (KBr) peaks: 2313, 1795 (ν_C=N_), 1518, 1376, 1251, 1080 (ν_C–N_), 840, 758, and 641 cm^−1^. ESI-MS: 458.96 [M]. CCDC No. 2259854.

5-chloro-2-*N*-(2-quinolylmethylene)aminophenoxide-Mn(II)-chloride/dihydrate (C3). Yield: 64.2%. Solubility in saline: ≥0.8 mg/mL. Melting points: 256 °C. Elemental analysis for C_16_H_14_Cl_2_MnN_2_O_3_·CH_3_OH: C, 46.39; H, 4.12; N, 6.36; O, 14.54. Found: C, 46.50; H, 4.09; N, 6.31; O, 14.48. IR spectrum of C3. IR (KBr) peaks: 2900 (ν_H–O–H_), 2314, 1741 (ν_C=N_), 1583, 1518, 1426, 1235, 1079 (ν_C–N_), 834, 754, and 690 cm^−1^. ESI-MS: 335.02 [M-2H_2_O-Cl]. CCDC No. 1033296.

### 3.3. The Stability of Cu, Zn, and Mn Complexes

These metal complexes were dissolved in saline (0.9% sodium chloride solution) and stored at room temperature for 0 h, 24 h, and 48 h, respectively, and their absorbance was determined by UV-vis spectroscopy.

### 3.4. Wound-Healing Assay

To evaluate the impact of C1 on cell migration, a wound-healing assay was carried out. A549 cells were seeded into 6-well plates and incubated overnight at 37 °C. C1 was dissolved in saline. Subsequently, scratch wounds were performed in the monolayer of cells using sterile toothpicks, and then the cells were treated with the Cu complex (2 μM and 4 μM). The cells were captured using an inverted microscope at 0 and 24 h.

### 3.5. Transwell Invasion Assay

The effect of C1 on tumor cell invasiveness was assessed by transwell assay. In brief, the transwell chamber was pre-coated with 70 μL diluted matrigel for 8 h at 37 °C. A549 cells were seeded to the upper chamber, and then C1 was added to the chamber. After 24 h of incubation, the chambers were washed, stained with 0.5% crystal violet for 20 min, and captured using an inverted microscope.

### 3.6. Cell Apoptosis Analysis

The A549 cells were placed in 6-well plates and incubated overnight. The cells were treated with C1 (4 μM and 8 μM) and incubated for 24 h. Subsequently, the cells were harvested and stained with Annexin V/PI for 20 min. The samples were detected by flow cytometry.

### 3.7. Mitochondrial Membrane Potential (ΔΨm) Assay

Exponentially growing A549 cells were cultured in 6-well plates. Subsequently, C1 (4 μM and 8 μM) was exposed to the cells for 24 h. The cells were stained with JC-1 probe and analyzed by flow cytometry.

### 3.8. Cell Cycle Analysis

The A549 cells were seeded into 10 cm dishes and incubated overnight. Subsequently, the cells were treated with C1 (4 μM and 8 μM). After 48 h, the cells were collected, fixed with 75% ethanol overnight, stained with PI for 20 min, and measured by flow cytometry.

### 3.9. β-Galactosidase Staining Assay

A549 cells were cultured in 6-well plates and incubated overnight. After C1 treatment for 24 h, the cells were fixed. Then, 20 min later, the cells were washed and stained with β-galactosidase for 48 h. The stained cells were observed and captured using an inverted microscope.

### 3.10. Measurement of Cellular ROS

The ROS in A549 cells was analyzed by flow cytometry. After pretreatment with C1 (4 μM and 8 μM) for 24 h, the A549 cells were collected and stained with H_2_DCF-DA. After 20 min, the samples were measured using a flow cytometer.

### 3.11. Comet Assay

The A549 cells were seed into 6-well plates and treated with C1. A mixture of 10 μL cell suspension and 70 μL low-melting-point agarose (1%) were placed on a glass microscope slide (pre-coated with 1% normal-melting-point agarose) and covered with coverslips. The cells were separate by gel electrophoresis, stained with PI, and captured using an inverted fluorescence microscope.

### 3.12. Fluorescence-Quenching Assay

The fluorescence spectrum was performed to measure the interaction mode of C1 (0−16 μM) and EB-DNA (8 μM ethidium bromide added into 10 μM CT-DNA) in a pH 7.2 Tris–HCl buffer. The EB-DNA was excited at 510 nm, and for the emission spectra, the emission range was set to 530–750 nm. C1 was gradually added to the EB-DNA solution.

### 3.13. Molecular Docking Study

The molecular docking method was performed using AutoDock 4.2. Docking studies were determined using MGL tools with AutoGrid and AutoDock to perform blind docking calculations between both C1 and DNA. The results were analyzed using Pymol software.

### 3.14. In Vivo Antitumor Experiment

BALB/C nude mice (6–8 weeks old, 18–22 g) were purchased from Hunan SJA Laboratory Animal Co., Ltd. An amount of 200 μL of A549 cells (containing 2 × 10^6^ cells) were subcutaneously injected into the left flank of the mice. The mice were randomly divided into 3 groups when the average tumor size reached 100 mm^3^. Then, the mice were treated with saline and C1 (2.5 and 5 mg/kg) for two weeks. C1 was dissolved in saline. During the treatment, the tumor volume and body weight of mice were measured every two days. The major organs (heart, kidney, and liver) and tumor tissues were harvested and sliced for H&E staining and TUNEL staining, respectively.

## 4. Conclusions

In summary, we designed and synthesized Cu(II), Zn(II), and Mn(II) complexes derived from 5-chloro-2-*N*-(2-quinolylmethylene)aminophenol. Indeed, the Cu complex exhibited effective cytotoxicity toward lung cancer cells and remarkably inhibited A549 cell migration. C1 also displayed an outstanding inhibitory effect against A549 tumor growth in vivo. Furthermore, we confirmed the mechanism of tumor growth inhibition by inducing mitochondrial apoptosis, interacting with DNA, blocking cell cycle arrest, inducing cell senescence, and inducing DNA damage.

## Figures and Tables

**Table 1:** IC_50_ (μM) values of Cu(II), Zn(II), and Mn(II) complexes against various cell lines.

Compound	A549	MCF-7	Hela	A549cisR	WI-38	SI ^a^
L	>40	>40	>40	>40	>40	-
C1	3.93 ± 0.28	5.30 ± 0.35	6.31 ± 0.32	6.79 ± 0.38	10.22 ± 0.43	2.60
C2	18.26 ± 0.52	23.61 ± 0.98	22.75 ± 0.80	29.33 ± 0.85	33.96 ± 1.06	1.86
C3	33.61 ± 0.98	>40	>40	>40	>40	-
Cisplatin	12.54 ± 0.45	14.53 ± 0.48	15.33 ± 0.51	>40	9.61 ± 0.58	0.77

^a^ Selectivity index (SI) is determined as IC_50_ (WI-38)/IC_50_ (A549).

## Data Availability

Data will be made available upon request.

## Supplementary Materials

The following supporting information can be downloaded at: https://www.mdpi.com/article/10.3390/molecules28124876/s1, Figures S1–S12: The IR, NMR, and ESI-MS of ligand and complexes; Figure S13: UV-vis spectra of complexes C1–C3. Table S1: Crystal data and structure refinement for C1–C3; Table S2: Bond lengths/Å and bond angles/° for C1–C3. The complete crystallography data of C1–C3 can be obtained free of charge from the Cambridge Crystallographic Data Centre via www.ccdc.cam.ac.uk/structures, accessed on 30 May 2023, with CCDC numbers of 1436726 (for C1), 2259854 (for C2), and 1033296 (for C3).

## Sample Availability

Samples of the compounds (C1–C3) are available from the authors.
