# Supplementary material for: Anticancer Activity and Mode of Action of Cu(II), Zn(II), and Mn(II) Complexes with 5-Chloro-2-N-(2-quinolylmethylene)aminophenol
Source: Molecules. 2023 Jun 20;28(12):4876. doi: 10.3390/molecules28124876 (PMC10300901; doi:10.3390/molecules28124876)

checkCIF/PLATON report

You have not supplied any structure factors. As a result the full set of tests cannot be run.

THIS REPORT IS FOR GUIDANCE ONLY. IF USED AS PART OF A REVIEW PROCEDURE FOR PUBLICATION, IT SHOULD NOT REPLACE THE EXPERTISE OF AN EXPERIENCED CRYSTALLOGRAPHIC REFEREE.

No syntax errors found. CIF dictionary Interpreting this report

Datablock: 0922-yf-jm-i1310

Bond precision:	C-C = 0.0061 A		Wavelength=0.71073
Cell:	a=14.3656(10)	b=14.9057(8)	c=6.8654(4)
	alpha=90	beta=90.238(6)	gamma=90
Temperature:	296 K		

	Calculated	Reported
Volume	1470.07(16)	1470.07(16)
Space group	P 21/c	P 1 21/c 1
Hall group	-P 2ybc	-P 2ybc
Moiety formula	C16 H10 Cl2 Cu N2 O	C16 H10 Cl2 Cu N2 O
Sum formula	C16 H10 Cl2 Cu N2 O	C16 H10 Cl2 Cu N2 O
Mr	380.71	380.70
Dx,g cm-3	1.720	1.720
Z	4	4
Mu (mm-1)	1.850	1.850
F000	764.0	764.0
F000'	766.65	
h,k,lmax	17,17,8	17,17,8
Nref	2585	2581
Tmin,Tmax	0.636,0.704	0.803,1.000
Tmin'	0.623	

Correction method= # Reported T Limits: Tmin=0.803 Tmax=1.000
AbsCorr = MULTI-SCAN

Data completeness= 0.998 Theta(max)= 25.000

R(reflections)= 0.0461(1898) wR2(reflections)= 0.0933(2581)

S = 1.076 Npar= 199

The following ALERTS were generated. Each ALERT has the format
test-name_ALERT_alert-type_alert-level.
Click on the hyperlinks for more details of the test.

Alert level C

```
PLAT341_ALERT_3_C Low Bond Precision on  C-C Bonds ...............    0.00612 Ang.
```

Alert level G

```
PLAT004_ALERT_5_G Polymeric Structure Found with Maximum Dimension          1 Info
PLAT012_ALERT_1_G No      _shelx_res_checksum Found in CIF ......     Please Check
PLAT093_ALERT_1_G No s.u.'s on H-positions, Refinement Reported as      mixed Check
PLAT720_ALERT_4_G Number of Unusual/Non-Standard Labels ..........         26 Note
PLAT794_ALERT_5_G Tentative Bond Valency for Cu1       (II)      .       2.11 Info
PLAT967_ALERT_5_G Note: Two-Theta Cutoff Value in Embedded .res ..       50.0 Degree
```

0 **ALERT level A** = Most likely a serious problem - resolve or explain
0 **ALERT level B** = A potentially serious problem, consider carefully
1 **ALERT level C** = Check. Ensure it is not caused by an omission or oversight
6 **ALERT level G** = General information/check it is not something unexpected

2 ALERT type 1 CIF construction/syntax error, inconsistent or missing data
0 ALERT type 2 Indicator that the structure model may be wrong or deficient
1 ALERT type 3 Indicator that the structure quality may be low
1 ALERT type 4 Improvement, methodology, query or suggestion
3 ALERT type 5 Informative message, check

It is advisable to attempt to resolve as many as possible of the alerts in all categories. Often the minor alerts point to easily fixed oversights, errors and omissions in your CIF or refinement strategy, so attention to these fine details can be worthwhile. In order to resolve some of the more serious problems it may be necessary to carry out additional measurements or structure refinements. However, the purpose of your study may justify the reported deviations and the more serious of these should normally be commented upon in the discussion or experimental section of a paper or in the "special_details" fields of the CIF. checkCIF was carefully designed to identify outliers and unusual parameters, but every test has its limitations and alerts that are not important in a particular case may appear. Conversely, the absence of alerts does not guarantee there are no aspects of the results needing attention. It is up to the individual to critically assess their own results and, if necessary, seek expert advice.

Publication of your CIF in IUCr journals

A basic structural check has been run on your CIF. These basic checks will be run on all CIFs submitted for publication in IUCr journals (*Acta Crystallographica*, *Journal of Applied Crystallography*, *Journal of Synchrotron Radiation*); however, if you intend to submit to *Acta Crystallographica Section C* or *E* or *IUCrData*, you should make sure that full publication checks are run on the final version of your CIF prior to submission.

Publication of your CIF in other journals

Please refer to the *Notes for Authors* of the relevant journal for any special instructions relating to CIF submission.

PLATON version of 28/11/2022; check.def file version of 28/11/2022

Datablock 0922-yf-jm-i1310 - ellipsoid plot

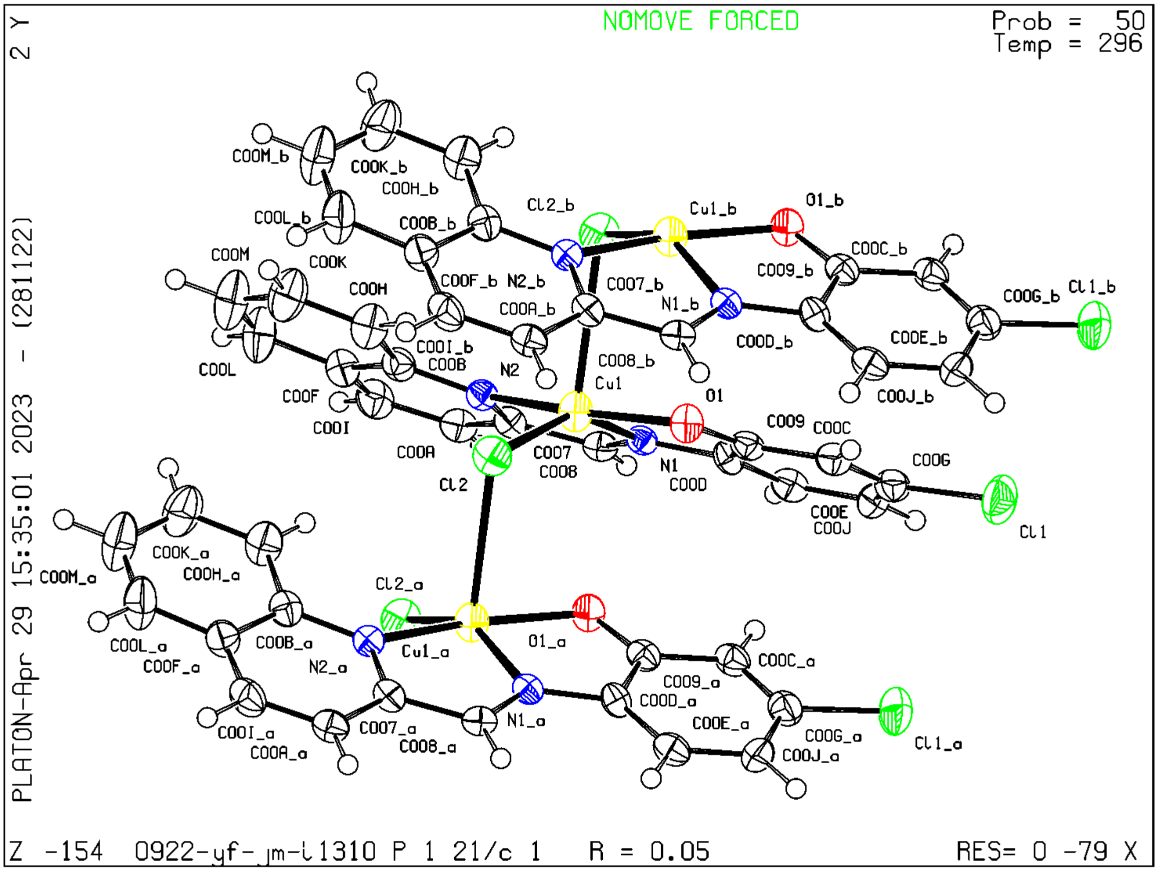

Supplement: Supplementary file 1 [file molecules-28-04876-s001.zip › checkcif-C1.pdf]
