# Supplementary material for: Anticancer Activity and Mode of Action of Cu(II), Zn(II), and Mn(II) Complexes with 5-Chloro-2-N-(2-quinolylmethylene)aminophenol
Source: Molecules. 2023 Jun 20;28(12):4876. doi: 10.3390/molecules28124876 (PMC10300901; doi:10.3390/molecules28124876)

checkCIF/PLATON report

You have not supplied any structure factors. As a result the full set of tests cannot be run.

THIS REPORT IS FOR GUIDANCE ONLY. IF USED AS PART OF A REVIEW PROCEDURE FOR PUBLICATION, IT SHOULD NOT REPLACE THE EXPERTISE OF AN EXPERIENCED CRYSTALLOGRAPHIC REFEREE.

No syntax errors found. CIF dictionary Interpreting this report

Datablock: ov_1124-yf-jm-ii06

```
Bond precision:   C-C = 0.0112 A          Wavelength=0.71073

Cell:             a=9.607(5)         b=10.533(6)       c=11.070(7)
                  alpha=79.41(5)     beta=64.70(6)     gamma=87.98(4)
Temperature:      296 K

                 Calculated                    Reported
Volume           994.3(11)                     994.3(10)
Space group      P -1                          P -1
Hall group       -P 1                          -P 1
Moiety formula   C21 H15 Cl2 N3 O Zn           C21 H15 Cl2 N3 O Zn
Sum formula      C21 H15 Cl2 N3 O Zn           C21 H15 Cl2 N3 O Zn
Mr               461.65                        461.63
Dx,g cm-3        1.542                         1.542
Z                2                             2
Mu (mm-1)        1.520                         1.520
F000             468.0                         468.0
F000'            469.28
h,k,lmax         11,12,13                      11,12,13
Nref             3512                          3451
Tmin,Tmax        0.723,0.761                   0.731,0.771
Tmin'            0.709

Correction method= # Reported T Limits: Tmin=0.731 Tmax=0.771
AbsCorr = ?

Data completeness= 0.983           Theta(max)= 25.020

R(reflections)= 0.0751( 2072)            wR2(reflections)= 0.2492( 3451)

S = 1.009                  Npar= 253
```

The following ALERTS were generated. Each ALERT has the format

test-name_ALERT_alert-type_alert-level.

Click on the hyperlinks for more details of the test.

Alert level C

```
CRYSC01_ALERT_1_C The word below has not been recognised as a standard
            identifier.
            dull
PLAT052_ALERT_1_C Info on Absorption Correction Method   Not Given     Please Do !
PLAT193_ALERT_1_C Cell and Diffraction Temperatures Differ by ....          3 Degree
PLAT232_ALERT_2_C Hirshfeld Test Diff (M-X)  Zn1      --N        .        5.3 s.u.
PLAT341_ALERT_3_C Low Bond Precision on  C-C Bonds ..............     0.0112 Ang.
```

Alert level G

```
PLAT005_ALERT_5_G No Embedded Refinement Details Found  in the CIF     Please Do !
PLAT072_ALERT_2_G SHELXL First  Parameter in WGHT  Unusually Large       0.13 Report
PLAT199_ALERT_1_G Reported _cell_measurement_temperature ..... (K)        293 Check
PLAT720_ALERT_4_G Number of Unusual/Non-Standard Labels ..........          3 Note
PLAT794_ALERT_5_G Tentative Bond Valency for Zn1       (II)      .       1.89 Info
```

0 **ALERT level A** = Most likely a serious problem - resolve or explain
0 **ALERT level B** = A potentially serious problem, consider carefully
5 **ALERT level C** = Check. Ensure it is not caused by an omission or oversight
5 **ALERT level G** = General information/check it is not something unexpected

4 ALERT type 1 CIF construction/syntax error, inconsistent or missing data
2 ALERT type 2 Indicator that the structure model may be wrong or deficient
1 ALERT type 3 Indicator that the structure quality may be low
1 ALERT type 4 Improvement, methodology, query or suggestion
2 ALERT type 5 Informative message, check

It is advisable to attempt to resolve as many as possible of the alerts in all categories. Often the minor alerts point to easily fixed oversights, errors and omissions in your CIF or refinement strategy, so attention to these fine details can be worthwhile. In order to resolve some of the more serious problems it may be necessary to carry out additional measurements or structure refinements. However, the purpose of your study may justify the reported deviations and the more serious of these should normally be commented upon in the discussion or experimental section of a paper or in the "special_details" fields of the CIF. checkCIF was carefully designed to identify outliers and unusual parameters, but every test has its limitations and alerts that are not important in a particular case may appear. Conversely, the absence of alerts does not guarantee there are no aspects of the results needing attention. It is up to the individual to critically assess their own results and, if necessary, seek expert advice.

Publication of your CIF in IUCr journals

A basic structural check has been run on your CIF. These basic checks will be run on all CIFs submitted for publication in IUCr journals (*Acta Crystallographica*, *Journal of Applied Crystallography*, *Journal of Synchrotron Radiation*); however, if you intend to submit to *Acta Crystallographica Section C* or *E* or *IUCrData*, you should make sure that full publication checks are run on the final version of your CIF prior to submission.

Publication of your CIF in other journals

Please refer to the *Notes for Authors* of the relevant journal for any special instructions relating to CIF submission.

PLATON version of 10/05/2023; check.def file version of 10/05/2023

Datablock ov_1124-yf-jm-ii06 - ellipsoid plot

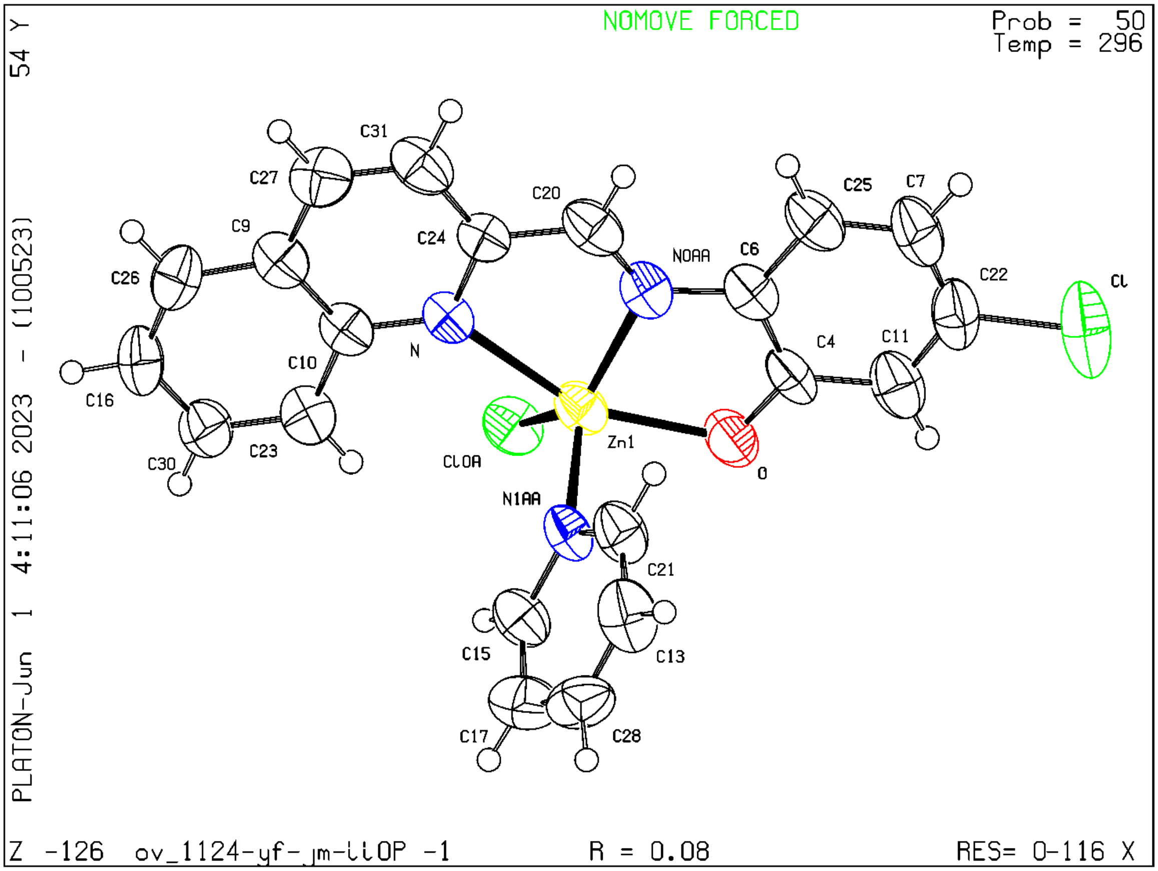

Supplement: Supplementary file 1 [file molecules-28-04876-s001.zip › checkcif-C2.pdf]
