# Supplementary material for: Anticancer Activity and Mode of Action of Cu(II), Zn(II), and Mn(II) Complexes with 5-Chloro-2-N-(2-quinolylmethylene)aminophenol
Source: Molecules. 2023 Jun 20;28(12):4876. doi: 10.3390/molecules28124876 (PMC10300901; doi:10.3390/molecules28124876)
Supplement: Supplementary file 1 [file molecules-28-04876-s001.zip › molecules-2414837-supplementary.pdf]

**Table S1.** Crystal data and structure refinement for C1-C3.

| Complexes | C1 | C2 | C3 |
|---|---|---|---|
| Empirical formula | $C_{16}H_{10}Cl_2CuN_2O$ | $C_{21}H_{15}Cl_2N_3OZn$ | $C_{17}H_{18}Cl_2MnN_2O_4$ |
| Formula weight | 380.70 | 461.63 | 440.17 |
| Crystal system | monoclinic | triclinic | triclinic |
| Space group | $P2_1/c$ | P-1 | P-1 |
| a/Å | 14.3656(10) | 9.607(5) | 7.080(4) |
| b/Å | 14.9057(8) | 10.533(6) | 11.346(6) |
| c/Å | 6.8654(4) | 11.070(7) | 13.326(7) |
| α/° | 90.00 | 79.41(5) | 107.037(8) |
| β/° | 90.238(6) | 64.70(6) | 100.842(9) |
| γ/° | 90.00 | 87.98(4) | 105.105(9) |
| Volume/Å$^3$ | 1470.07(16) | 994.3(10) | 946.7(9) |
| μ/mm$^{-1}$ | 1.850 | 1.520 | 1.004 |
| F(000) | 764.0 | 468.0 | 450.0 |
| Reflections collected | 6906 | 7331 | 11247 |
| Goodness-of-fit on $F^2$ | 1.076 | 1.009 | 0.959 |
| Final R indexes [I>=2σ (I)] | $R_1$ = 0.0461, $wR_2$ = 0.0869 | $R_1$ = 0.0751, $wR_2$ = 0.1902 | $R_1$ = 0.0459, $wR_2$ = 0.1099 |
| Final R indexes [all data] | $R_1$ = 0.0715, $wR_2$ = 0.0933 | $R_1$ = 0.1161, $wR_2$ = 0.2492 | $R_1$ = 0.0899, $wR_2$ = 0.1350 |

**Table S2.** Bond lengths /Å and bond angles /° for C1-C3.

| | | | |
|---|---|---|---|
| | | C1 | |
| Cu1-Cl2 | 2.2479(11) | O1-Cu1-Cl2 | 97.10(9) |
| Cu1-O1 | 1.959(3) | O1-Cu1-N2 | 162.74(12) |
| Cu1-N1 | 1.944(3) | N1-Cu1-Cl2 | 148.78(10) |
| Cu1-N2 | 2.076(3) | N1-Cu1-O1 | 83.90(12) |
| N2-Cu1-Cl2 | 99.79(9) | N1-Cu1-N2 | 79.82(13) |
| | | C2 | |
| Cl2-Zn | 2.225(2) | N2-Zn-Cl2 | 98.25(14) |
| N2-Zn | 2.365(5) | N3-Zn-O1 | 91.4(2) |
| N3-Zn | 2.077(6) | N3-Zn-N2 | 95.2(2) |
| O1-Zn | 2.066(5) | N1-Zn-Cl2 | 134.60(18) |
| N1-Zn | 2.061(6) | N1-Zn-O1 | 80.0(2) |
| N2-Zn-O1 | 153.16(19) | N1-Zn-N2 | 73.3(2) |
| N3-Zn-Cl2 | 115.96(16) | N1-Zn-N3 | 110.2(2) |
| | | C3 | |
| Mn1-Cl2 | 2.5214(18) | O1-Mn1-Cl2 | 95.59(8) |
| Mn1-O1 | 2.187(3) | O1-Mn1-O2 | 90.69(11) |
| Mn1-O3 | 2.163(3) | O1-Mn1-N1 | 73.98(10) |
| Mn1-O2 | 2.249(4) | O1-Mn1-N2 | 145.92(10) |
| Mn1-N1 | 2.219(3) | O3-Mn1-Cl2 | 84.59(10) |
| Mn1-N2 | 2.364(3) | O3-Mn1-O1 | 97.30(11) |
| O2-Mn1-N2 | 86.00(11) | O3-Mn1-O2 | 87.13(13) |
| N1-Mn1-Cl2 | 99.51(8) | O3-Mn1-N1 | 170.61(12) |
| N1-Mn1-O2 | 89.46(11) | O3-Mn1-N2 | 116.35(12) |
| N1-Mn1-N2 | 72.08(11) | O2-Mn1-Cl2 | 170.18(9) |
| N2-Mn1-Cl2 | 92.95(8) | | |

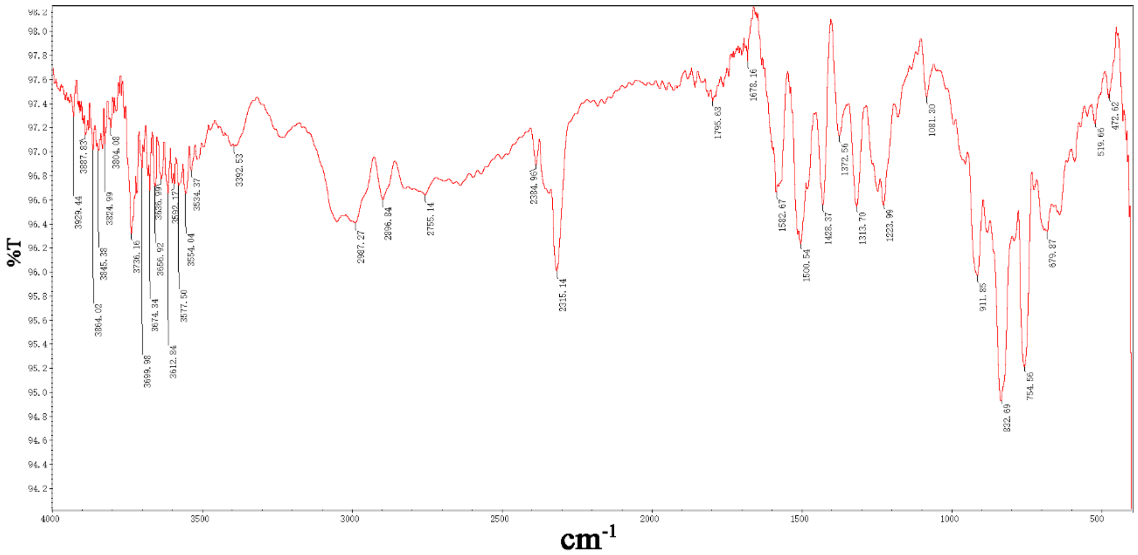

**Figure S1.** IR spectrum of L.

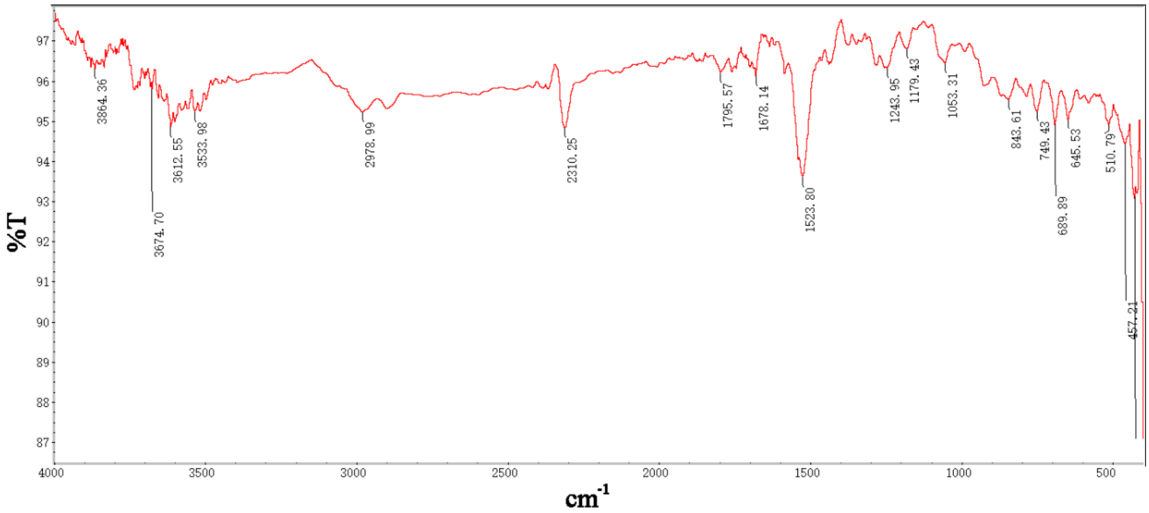

**Figure S2.** IR spectrum of C1.

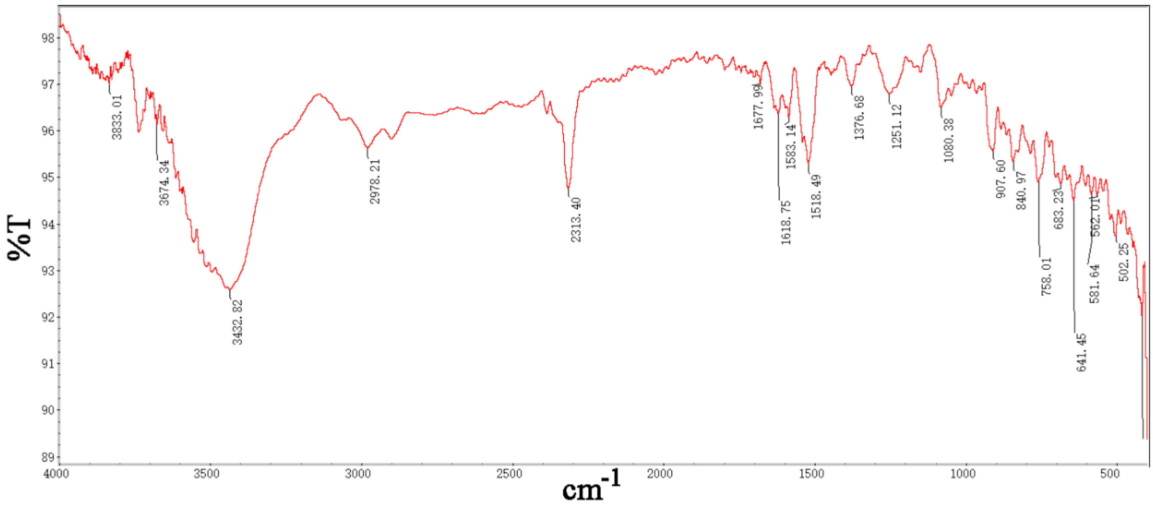


**Figure S3.** IR spectrum of C2.

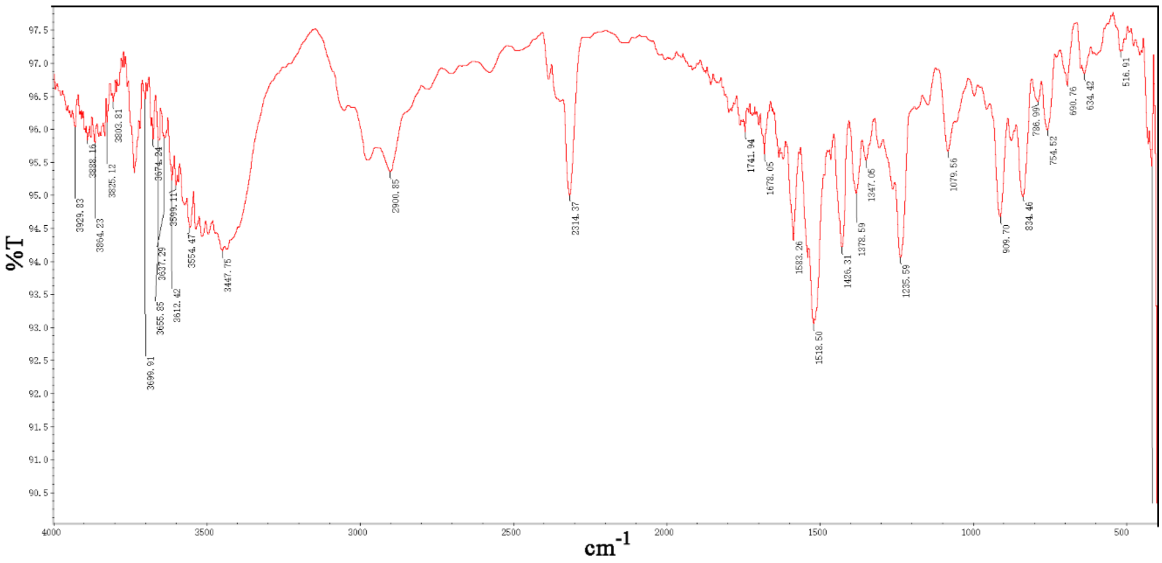


**Figure S4.** IR spectrum of C3.

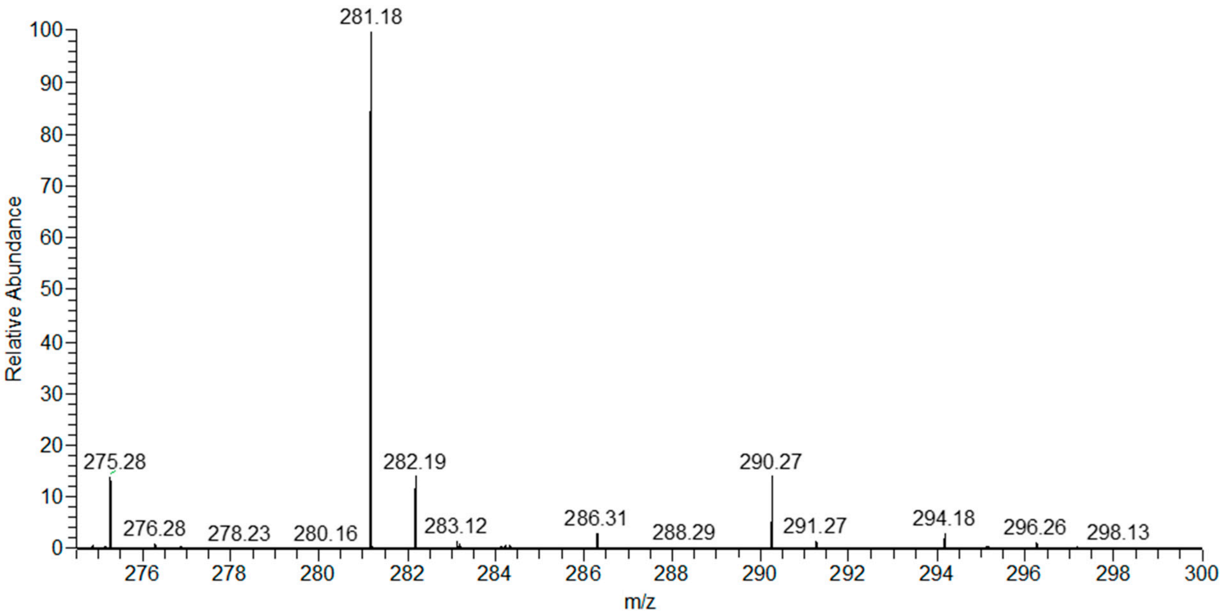


**Figure S5.** The ESI-MS of L.

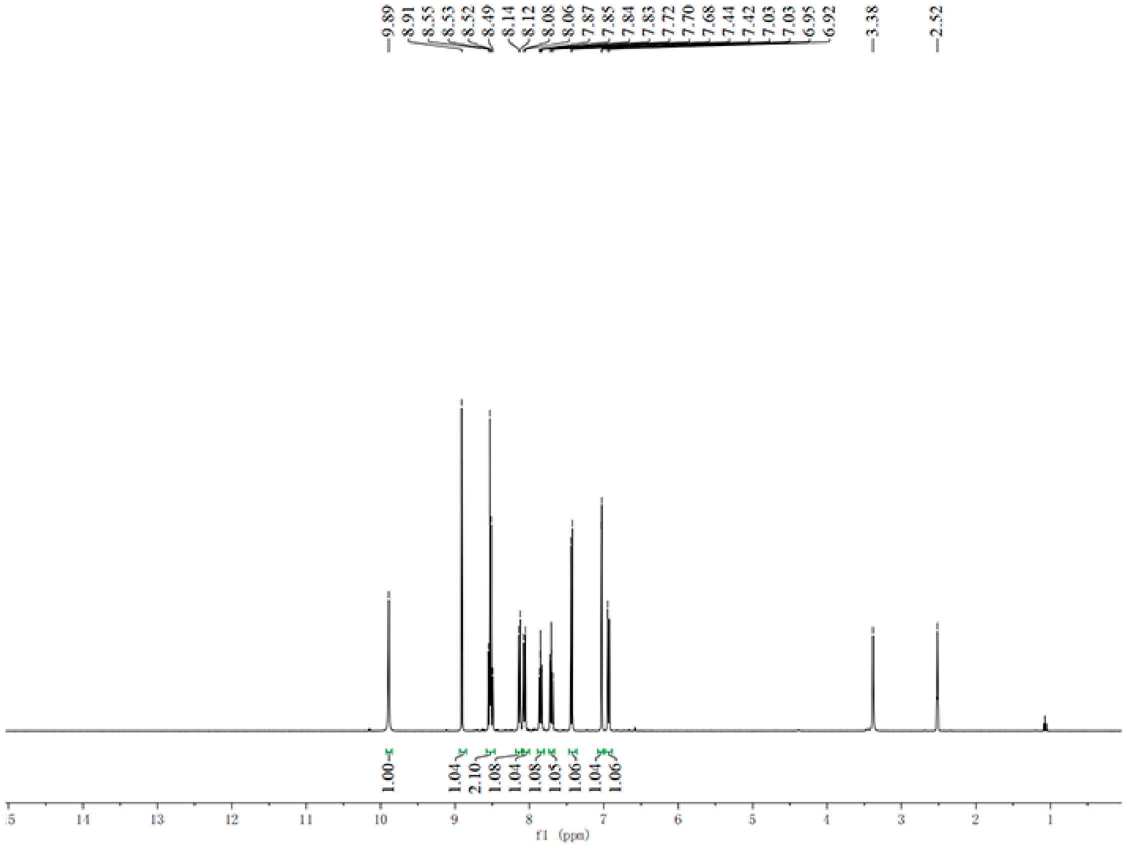


**Figure S6.** $^{1}$H-NMR spectrum of L.

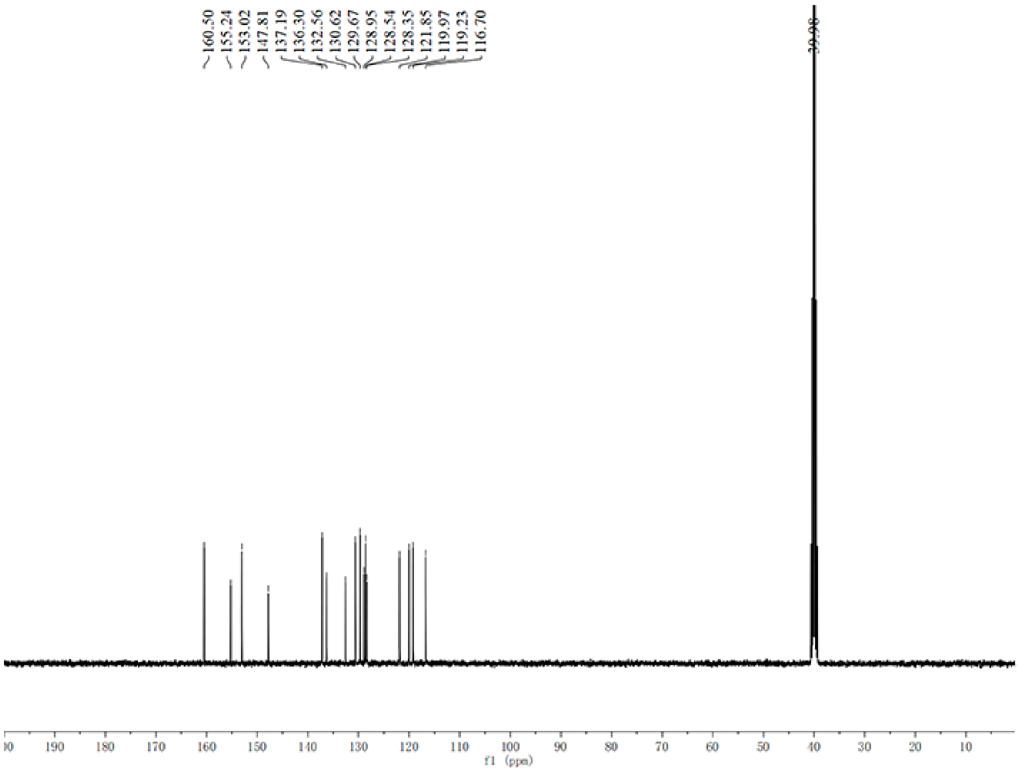


**Figure S7.** $^{13}$C-NMR spectrum of L.

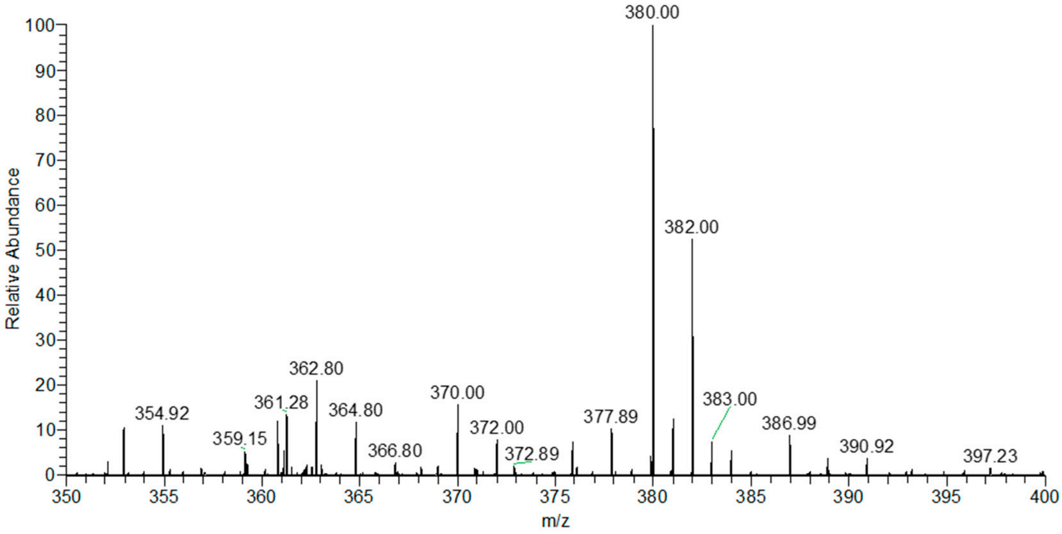


**Figure S8.** The ESI-MS of C1.

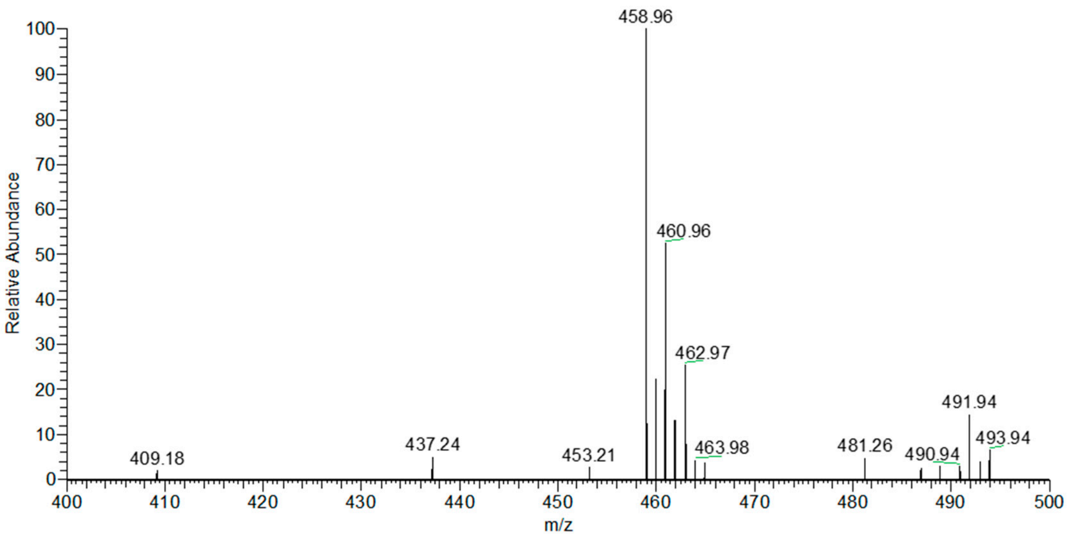


**Figure S9.** The ESI-MS of C2.

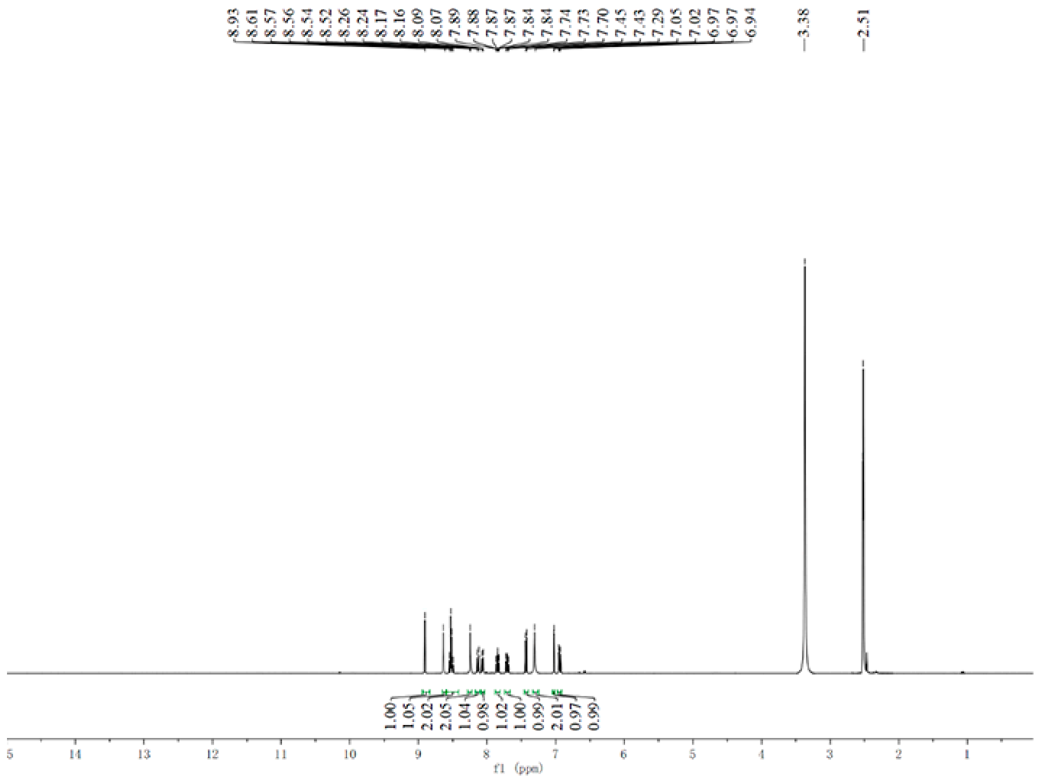


**Figure S10.** $^{1}$H-NMR spectrum of C2.

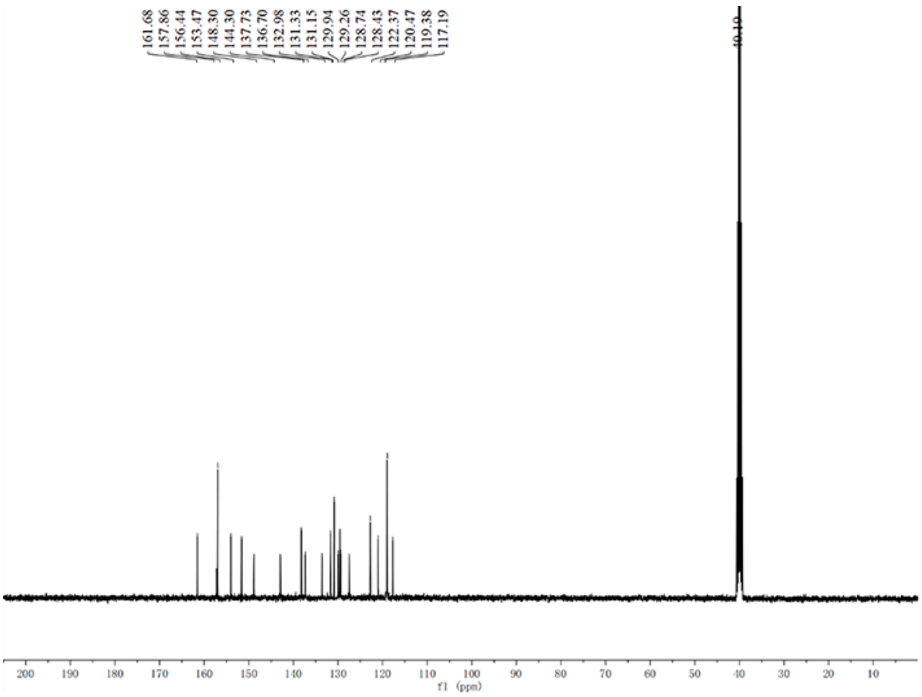


**Figure S11.** $^{13}$C-NMR spectrum of C2.

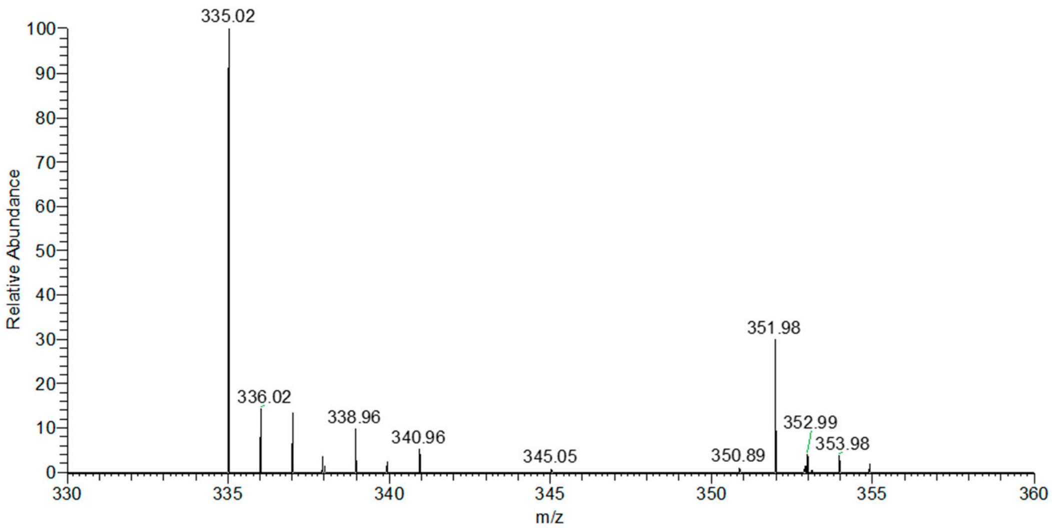


**Figure S12.** The ESI-MS of C3.

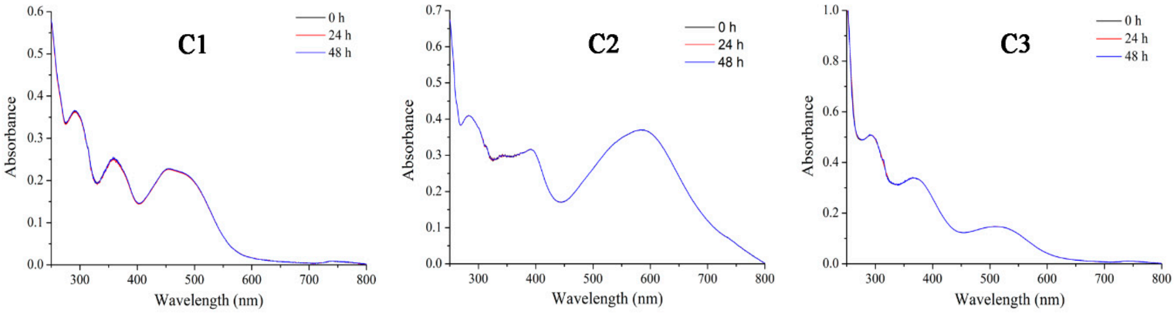


**Figure S13.** UV-vis spectra of complexes C1-C3 in saline.
